# Hsa_circ_0006006 is a potential biomarker for prognosis and cisplatin resistance in non-small cell lung cancer

**DOI:** 10.1186/s41065-025-00392-w

**Published:** 2025-03-08

**Authors:** Min Ding, Jing Zhao, XiaoNa Li

**Affiliations:** 1 Department of Oncology, Wuhan Third Hospital·Tongren Hospital of Wuhan University, Wuhan City, Hubei Province 430060 China; 2 Department of Pathology, The First Affiliated Hospital of Naval Military Medical University, Shanghai City, 200433 China; 3 Department of Pharmacy, Gaoling Hospital, No. 555 Shanglin 2nd Road, Gaoling District, Xi’an City, Shannxi Province 710200 China

**Keywords:** Non-small cell lung cancer, Cisplatin, Drug resistance, Hsa_circ_0006006, Prognosis, TNM stage

## Abstract

**Background and objective:**

Platinum-based drugs, such as cisplatin (DDP), are the standard treatment, yet drug resistance has become a key challenge. Previous studies have shown that hsa_circ_0006006 promotes non small cell lung cancer (NSCLC) progression. This study aimed to reveal the role of specific circRNAs in DDP resistance in NSCLC and their potential clinical applications.

**Methods:**

CircRNA sequencing data of three NSCLC tissue and three normal tissue samples were extracted from the GEO database based on conditions that matched the microarray expression profiles of circRNAs from human NSCLC lung samples and matched neighboring samples and raw matrix data and platform annotation data, and differential expression analysis was performed using the R language. Log2 Fold change > 1 and *P* < 0.05 were labeled as differential genes. Serum samples were collected from 31 NSCLC patients and 21 DDP-resistant NSCLC patients. The Kaplan-Meier method was used to detect the correlation between circRNA levels and survival prognosis of NSCLC patients. The relationship between circRNAs and clinicopathological characteristics of patients was assessed by chi-square test. RT-qPCR was performed to detect the expression of key circRNAs associated with DDP drug resistance. circRNAs were analyzed by ROC curves to assess the diagnostic potential. A549 cells and A549/DDP cells were cultured to verify the effect of up- and down-regulation of hsa_circ_0006006 on DDP drug resistance in NSCLC cells using colony formation assay and flow cytometry.

**Results:**

Abnormally elevated hsa_circ_0006006 expression was closely associated with NSCLC survival prognosis as well as DDP resistance (*p* < 0.05) with good diagnostic efficacy (AUC for NSCLC = 0.91, *p* < 0.01; AUC for DDP resistant = 0.80, *p* = 0.00). This was further validated in the analysis of clinical samples (*p* < 0.05). Knockdown of hsa_circ_0006006 significantly reduced DDP resistance in NSCLC cells, while overexpression of hsa_circ_0006006 had the opposite effect (*p* < 0.05).

**Conclusion:**

NSCLC survival prognosis is associated with aberrant expression of hsa_circ_0006006, which regulates NSCLC cell proliferation and apoptosis and thus promotes DDP drug resistance. These findings provide potential targets for patient prognosis and assessment of biomarkers of response to DDP therapies that can be used to aid in early diagnosis and prognostic assessment, as well as new options for the future development of relevant small-molecule inhibitors or nucleic acid drugs.

## Introduction

Comprising three main subtypes—adenocarcinoma, squamous cell carcinoma, and large cell carcinoma—non-small cell lung cancer (NSCLC) is the most common lung cancer type. Each subtype differs in biological behavior and treatment response [1]. The treatment and prognosis of NSCLC depend on a variety of biomarkers and molecular features. EGFR mutations and ALK fusion genes are often found in adenocarcinomas and are essential markers for the application of targeted treatments such as gefitinib and crizotinib [2, 3]. The expression level of PDL1 is a significant determinant in immunotherapy selection [4]. Survival prognosis is closely related to tumor stage. Early NSCLC (stages I and II) has the potential to be cured through surgery and adjuvant therapy, whereas the five-year survival rate for patients with advanced stages (stages III and IV) is typically less than 10%. The survival time is largely dependent on the biology of the tumor and the response to therapy [5]. The challenge of cisplatin resistance in NSCLC treatment is tied to mechanisms including DNA repair, cellular exclusion of platinum drugs, inhibition of apoptosis, and modifications in the tumor microenvironment [6]. Among the DNA repair mechanisms, ERCC1 and BRCA are key proteins whose high expression is associated with platinum drug resistance [7, 8].

circRNAs are a type of non-coding RNAs involved in numerous biological processes, particularly in gene expression regulation [9]. In cancer, circRNAs affect tumorigenesis and progression through a variety of mechanisms. For example, they can act as miRNA “sponges” and inhibit miRNA function, thereby increasing the expression of miRNA target genes [10]. Due to their high stability and tissue specificity, circRNAs serve as excellent biomarkers [11]. In NSCLC, certain circRNA expression profiles can differentiate normal lung tissue from tumor tissue, potentially enabling early diagnosis [12]. For example, circSPECC1 and circ-SLC16A1 are both upregulated in NSCLC and inhibit tumor growth by suppressing cancer cell proliferation and metastasis [13, 14]. Additionally, specific circRNAs are associated with disease prognosis in NSCLC patients, providing new molecular markers for assessing prognosis [15, 16]. CircRNAs might also act as significant molecular markers for anticipating responses to chemotherapy and resistance to drugs [17, 18]. Inhibiting circPDK1, which is upregulated in NSCLC, enhances chemotherapy sensitivity [19]. However, circ 0006006 (circPDK1) has been little studied in NSCLC. circ_0006006 silencing inhibits the proliferative capacity, migration, invasion, and angiogenesis of NSCLC cells, and promotes apoptosis. This is also validated in an in vivo experiment [20]. This brought circ_0006006 to our attention, emphasizing the importance of exploring its link to NSCLC progression and drug resistance in the clinic.

The research was centered on identifying circRNAs that aid in clinical diagnosis, survival prognosis, and DDP resistance diagnosis of NSCLC through bioinformatics. It was hoped to personalize treatment strategies and improve treatment outcomes.

## Materials and methods

### Bioinformatic analysis

The GEO database provided open-access circRNA expression profiles and related information. Microarrays were searched using “lung cancer” or “lung tumor” and “circRNA” to fulfill the requirements of the GEO dataset. Inclusion and exclusion criteria were shown as follows (1) microarray expression profiles of circRNAs, (2) inclusion of human NSCLC lung samples and matched neighboring samples, and (3) raw matrix data and platform annotation data. GSE112214 was finally downloaded. We extracted circRNA expression profiles of three NSCLC tissue samples and three paracancerous normal tissue samples from the GSE112214 project. Differential genes in the database were analyzed by R language software. Genes with Log2 Fold change > 1 and *P* < 0.05 were labeled as differentially expressed genes. Gene information for hsa_circ_0006006 was retrieved from the bioinformatics website circbank (http://www.circbank.cn/index.html).

### Clinical sampling

During 2016–2018, serum samples from 31 patients with NSCLC and 19 healthy subjects were collected at Wuhan Third Hospital·Tongren Hospital of Wuhan University. Serum samples were collected from 48 NSCLC patients treated with DDP. All serum samples were rapidly frozen at -80 °C after collection for subsequent analysis. CT examination results of patients before and after DDP treatment were evaluated according to Response Evaluation Criteria in Solid Tumors 1.1 (RECIST 1.1). DDP-resistant NSCLC patients (12 patients) were defined as patients who developed recurrence after DDP treatment. DDP-sensitive patients (36 cases) demonstrated good progression without recurrence. All subjects read and fully understood the pre-signed written informed consent form, which clearly listed the key information about the study and the rights of the subjects. Anonymization was initiated at the data collection stage. Strict security measures were taken to protect the subjects’ relevant information and data during each data analysis and sharing. The study was approved by the Ethics Committee of Wuhan Third Hospital·Tongren Hospital of Wuhan University (No.201506TR23). Lymph node metastasis and tumor differences were assessed using MRI and CT, respectively. The tumors were graded and staged according to WHO grading criteria and UICC grading criteria (7th edition).

### Survival prognosis analysis

Over a five-year period, a cohort of 31 NSCLC patients was observed to assess their survival prognosis. Patients were contacted monthly for interviews, either by phone or in person, during the follow-up. The collected data were included in the survival analysis, and survival rates were calculated using the Kaplan-Meier method.

### RT-qPCR

Using Trizol reagent (Life technologies), total RNA was collected from cells and tissues, and the quality of RNA was measured with a NanoDrop 2000 spectrophotometer (Thermo Fisher Scientific, USA). The process of reverse transcribing to cDNA utilized the PrimeScript RT Master Mix (Takara; RR036A). The identification of cDNA was achieved through ChamQ SYBR qPCR Master Mix (Vazyme; Q311-02), followed by sequencing using the Roter Gene 3000 Sequence Detection System (Corbett Research, Australia). The comparative expression of genes was determined using the 2^−ΔΔCt^ technique and standardized against GAPDH levels. The primers are shown in Table 1.

**Table 1:** Primer sequence

Gene	Forward primer (5′ →3′)	Reverse primer (5′ →3′)
hsa_circ_0006006	AAAGACATGACGACGTTCCG	TACCCAGCGTGACATGAACT
hsa_circ_0017109	ACACGGAGCTGTACCACTAC	TGCTCCCCACAAAGATCCTT
hsa_circ_0007580	GCTGACTTTGGGATGTGCA	GGCAAGCATCACCTTTCCAA
hsa_circ_0017956	CAACACATCCAATGCCAGCT	CACTGCCTCTCCAAAAGCTG
hsa_circ_0072309	TTCCACACCGCTCAAATGTT	AGCCACTGGAAATTTGAAGCA
hsa_circ_0008234	ACAGCTCTCAGTCCACACTC	TGAAGCTGCAACTGTTCCTG
hsa_circ_0006677	AGGGCAGTTTACAAGGTCAGT	AAGCACGGTTTTGGACACAG
hsa_circ_0001947	CGGACATCTCACCAACACTG	TTTCCAAGCGTGTTCTGGAC
hsa_circ_0072305	GCTCATCACCACCTTCCAAA	AGCCACTGGAAATTTGAAGCA
GAPDH	GTCAAGGCTGAGAACGGGAA	AAATGAGCCCCAGCCTTCTC

### Cell culture

Human NSCLC cell line (A549) and DDP-resistant cell line (A549/DDP) sourced from Chinese Academy of Sciences (Shanghai, China) were cultured in DMEM (Gibco) containing 10% fetal bovine serum (10099-141; Gibco), 100 U/ml penicillin, and 100 µg/ml streptomycin (YEASEN, Shanghai, China). Cells were incubated in a humidified atmosphere of 5% CO_2_ at 37 °C. Drug-resistant cells were sustained in a medium containing 2 mg/L DDP (P4394; Sigma-Aldrich). Cells were grown to 80% at 1:3 passaging culture, and logarithmic growth phase A549 cells or A549/DDP cells after 2 passages were taken for experimental studies.

### Actinomycin D assay and RNase R assay

A549 cells were inoculated in six-well plates (5 × 10^5^ cells/well). After 24 h, cells were exposed to 2 µg/ml actinomycin D (Sigma) and collected at the indicated time points. The stability of hsa_circ_0006006 and GAPDH mRNA was analyzed using RT-qPCR.

RNase R (3 U/g, Epicenter) was used to treat RNA extracted from A549 cells (10 µg) at 37 °C for 30 min. Then, hsa_circ_0006006 and GAPDH were detected using RT-qPCR.

### Cell transfection

The siRNA and pcDNA 3.1 overexpression plasmid targeting hsa_circ_0006006 and the corresponding negative control (GenePharma, Shanghai, China) were transiently transfected into A549/DDP cells using Lipofectamine 3000 (Invitrogen). After 48 h, transfection efficiency was assessed by RT-qPCR.

### Colony formation assay

Transfected A549/DDP cells were treated with DDP at 8 mg/L for 24 h and cultured in 6-well plates at 500 cells/well for 14 days. Following this, the cells were fixed with methanol, cleansed twice using PBS, and then dyed with a 0.1% crystal violet solution (Beyotime, Shanghai, China). Using a Nikon microscope (Tokyo, Japan), colonies were examined and tallied across six fields of view.

### Flow cytometry

Transfected A549/DDP cells were treated with 8 mg/L DDP for 24 h. The Annexin V-FITC/PI Apoptosis Detection Kit (Vazyme) was employed to determine apoptosis. The cells were identified using a BD Accuri^®^ C6 Plus (BD Biosciences, NJ, USA) and examined through FlowJo. The cells were reconstituted in 100 µl of 1 × binding buffer and treated with 5 µl of Annexin V-FIFC and PI staining solution, followed by a 10-min incubation. Ultimately, a volume of 400 µl of 1 × binding buffer was introduced into the culture and subjected to flow cytometry analysis within an hour.

### Data analysis

Each experiment involved at least three biological duplicates, with the results presented as mean ± standard deviation (SD). The statistical evaluation was conducted utilizing GraphPad Prism 9.0 (GraphPad Software, USA). The diagnostic value of hsa_circ_0006006 for the detection of NSCLC and its DDP resistance was assessed using a receiver operating characteristics (ROC) curve to obtain the area under the curve (AUC), sensitivity, and specificity. The chi-square test was employed to assess the correlation between circRNAs and the clinicopathological characteristics of NSCLC patients. The association between circRNAs and survival was examined using Kaplan-meier survival curves. The Shapiro-Wilk test was utilized to evaluate the normal distribution pattern of the samples. The Student’s t-test facilitated comparisons between two groups, while one-way ANOVA was applied for numerous comparisons, succeeded by Tukey HSD. * *P*-value less than 0.05 was deemed to be statistically significant.

## Results

### Differential circrna profiles in NSCLC

**Fig. 1:**
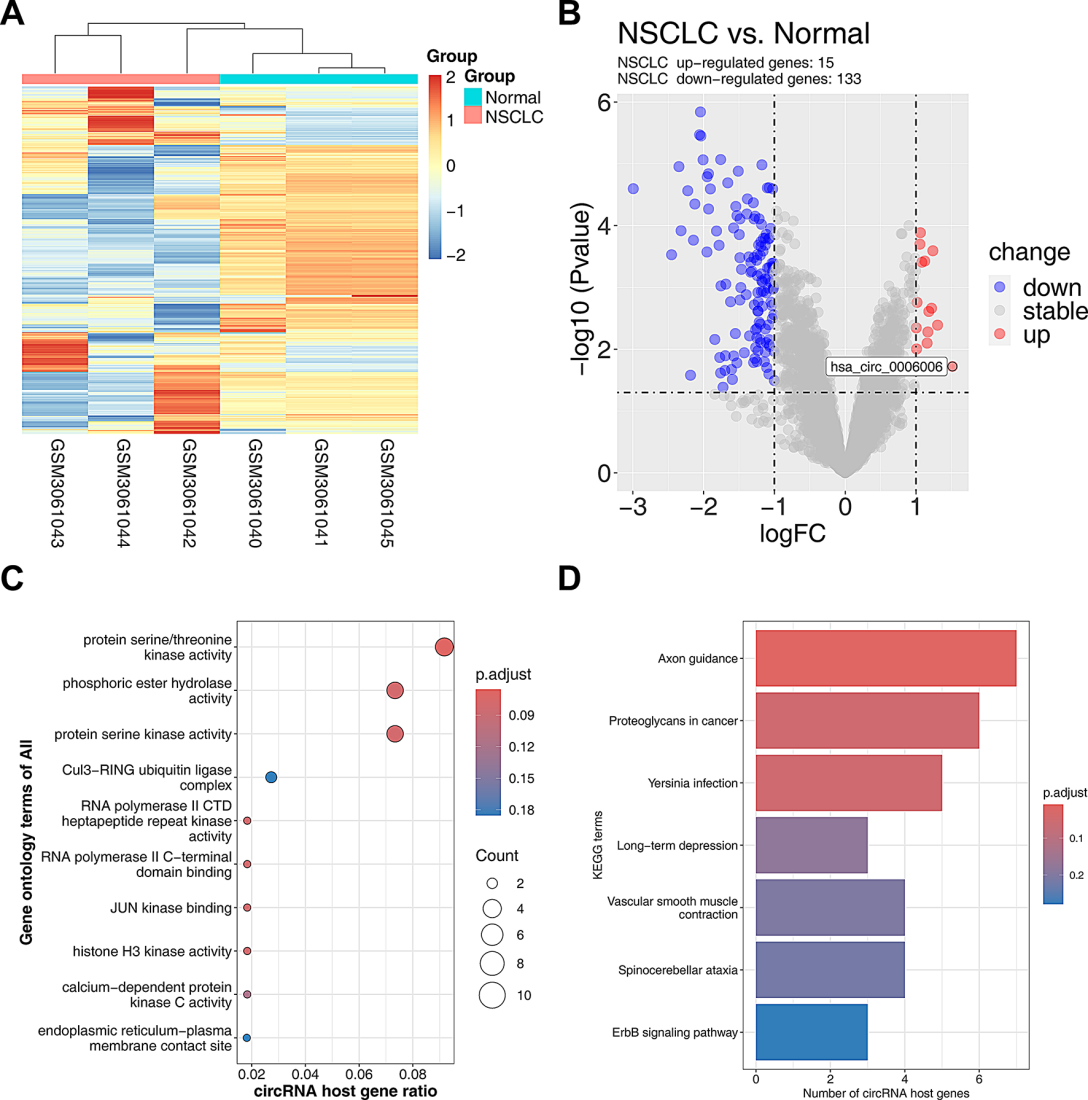
Differential circRNA profiles in NSCLC. **A** Heatmap showing the top 500 circRNA expression profiles in normal and NSCLC samples; **B** Volcano plot showing genes that were abnormally up-regulated and down-regulated in NSCLC tissues; **C** GO analysis of host genes of differentially expressed circRNAs; **D** KEGG enrichment pathway analysis of differentially expressed circRNAs

To explore the aberrantly expressed circRNAs in NSCLC, we selected GSE datasets (GSE112214) from the GEO database and performed expression profiling of differentially expressed circRNAs by R language software. The heatmap demonstrated the top 500 most abundant circRNAs (Fig. 1A). A total of 133 abnormally low-expressed circRNAs and 15 abnormally high-expressed circRNAs were identified in NSCLC tissues (Fig. 1B). We used GO and KEGG pathway enrichment analyses to elucidate the functions of disease-associated circRNAs and their roles in the bioregulatory network (Fig. 1C). GO analysis highlighted several key biological processes and molecular functions, such as the regulation of expression of the endoplasmic reticulum-plasma membrane contact site (ER-PM contact sites) bound to the cell membrane. In addition, enrichment of protein serine/threonine kinase activity revealed the central role of this kinase class in regulating cell cycle and proliferation. KEGG pathway analyses further revealed the aberrant activation of several key biological pathways. Notable pathways included the ErbB signaling pathway, proteoglycans, vascular smooth muscle contraction, and chronic depression (Fig. 1D). The enrichment of these pathways emphasizes the potential value of targeted therapies.

### hsa_circ_0006006 is highly expressed in NSCLC while Hsa_circ_0008234 and Hsa_circ_0006677 are lowly expressed

**Fig. 2:**
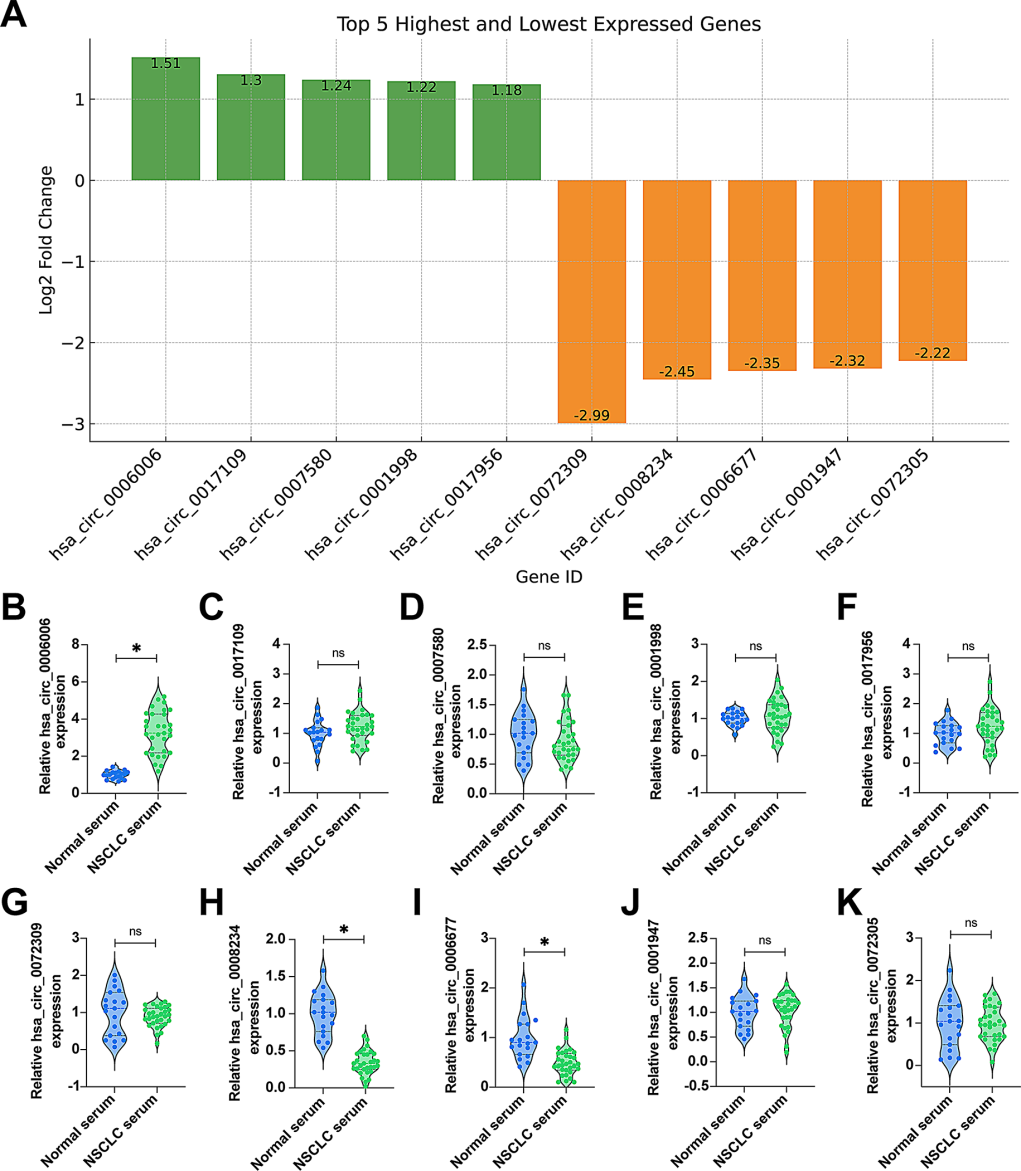
Expression analysis of differential circRNAs in the serum of patients. **A** Bar graphs showing the top 5 upregulated and downregulated circRNAs in GSE112214 database and Log2 FC values; **B**–**K** RT-qPCR assay of the top 5 upregulated and downregulated circRNAs in the serum of healthy controls and patients with NSCLC; data are expressed as mean ± SD (*N* = 3)

Subsequently, we analyzed the top 5 highest and lowest expressed circRNAs (Fig. 2A). To verify whether the sequencing data were consistent with the data from clinical samples, we performed RT-qPCR analysis of these 10 circRNAs. As shown in Fig. 2B-K, only hsa_circ_0006006, hsa_circ_0008234 and hsa_circ_0006677 were found to be expressed in NSCLC tissues (*P* < 0.05), and the remaining 7 circRNAs were found to be differentially expressed (*P* > 0.05).

### Correlation between Hsa_circ_0006006, Hsa_circ_0008234, and Hsa_circ_0006677 and the clinicopathologic parameters of patients

Using a chi-square test, the link between hsa_circ_0006006, hsa_circ_0008234, and hsa_circ_0006677 and the clinicopathologic features of NSCLC patients was examined. As shown in Tables 2, 3 and 4, hsa_circ_0006006 was correlated with TNM stage and lymph node metastasis in NSCLC patients. hsa_circ_0008234 was correlated with TNM stage in NSCLC patients. hsa_circ_0006677 was not correlated with clinicopathologic characteristics of NSCLC patients.

**Table 2:** The relationship between Hsa_circ_0006006 and clinicopathological features of NSCLC patients

Characteristic	Cases	The expression of hsa_circ_0006006		*P*
	*n* = 31	Low (*n* = 15)	High (*n* = 16)	
Gender				0.6109
Male	20	9	11	
Female	11	6	5	
Age (year)				0.2131
≤ 60	13	8	5	
> 60	18	7	11	
Tumor size				0.4744
< 3 cm	23	12	11	
≥ 3 cm	8	3	5	
TNM stage				0.0384*
I/II	19	12	7	
II/IV	12	3	9	
Lymph node metastasis				0.0451*
Positive	14	4	10	
Negative	17	11	6	
Distant metastasis				0.3205
Positive	11	4	7	
Negative	20	11	9	

**Table 3:** The relationship between Hsa_circ_0008234 and clinicopathological features of NSCLC patients

Characteristic	Cases	The expression of hsa_circ_0008234		*P*
	*n* = 31	Low (*n* = 15)	High (*n* = 16)	
Gender				0.2077
Male	20	8	12	
Female	11	7	4	
Age (year)				0.6052
≤ 60	13	7	6	
> 60	18	8	10	
Tumor size				0.1244
< 3 cm	23	13	10	
≥ 3 cm	8	2	6	
TNM stage				0.0020*
I/II	19	5	14	
II/IV	12	10	2	
Lymph node metastasis				0.8705
Positive	14	7	7	
Negative	17	8	9	
Distant metastasis				0.3205
Positive	11	4	7	
Negative	20	11	9	

**Table 4:** The relationship between Hsa_circ_0006677 and clinicopathological features of NSCLC patients

Characteristic	Cases	The expression of hsa_circ_0006677		*P*
	*n* = 31	Low (*n* = 15)	High (*n* = 16)	
Gender				0.3205
Male	20	11	9	
Female	11	4	7	
Age (year)				0.2131
≤ 60	13	8	5	
> 60	18	7	11	
Tumor size				0.0803
< 3 cm	23	9	14	
≥ 3 cm	8	6	2	
TNM stage				0.1826
I/II	19	11	8	
II/IV	12	4	8	
Lymph node metastasis				0.1080
Positive	14	9	5	
Negative	17	6	11	
Distant metastasis				0.6109
Positive	11	6	5	
Negative	20	9	11	

### hsa_circ_0006006 is associated with poor prognosis in NSCLC

**Fig. 3:**
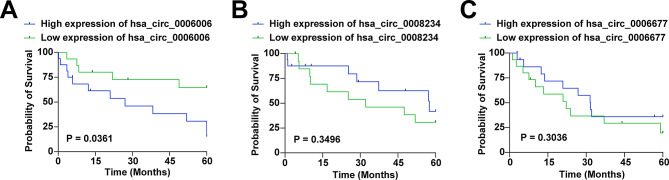
hsa_circ_0006006 is associated with poor prognosis in NSCLC. **A**–**C** The association of hsa_circ_0006006, hsa_circ_0008234 and hsa_circ_0006677 with survival prognosis of NSCLC patients was analyzed using Kaplan-Meier test

By Kaplan-Meier method, we evaluated the association of hsa_circ_0006006, hsa_circ_0008234 and hsa_circ_0006677 with survival prognosis of NSCLC patients. NSCLC patients with high expression of hsa_circ_0006006 had worse survival prognosis (*P* = 0.04). hsa_circ_0006006 (*P* = 0.35) and hsa_circ_0008234 (*P* = 0.30) did not correlate with the survival prognosis of NSCLC patients (Fig. 3A-C).

### hsa_circ_0006006 and Hsa_circ_0008234 May serve as clinical diagnostic markers for NSCLC

**Fig. 4:**
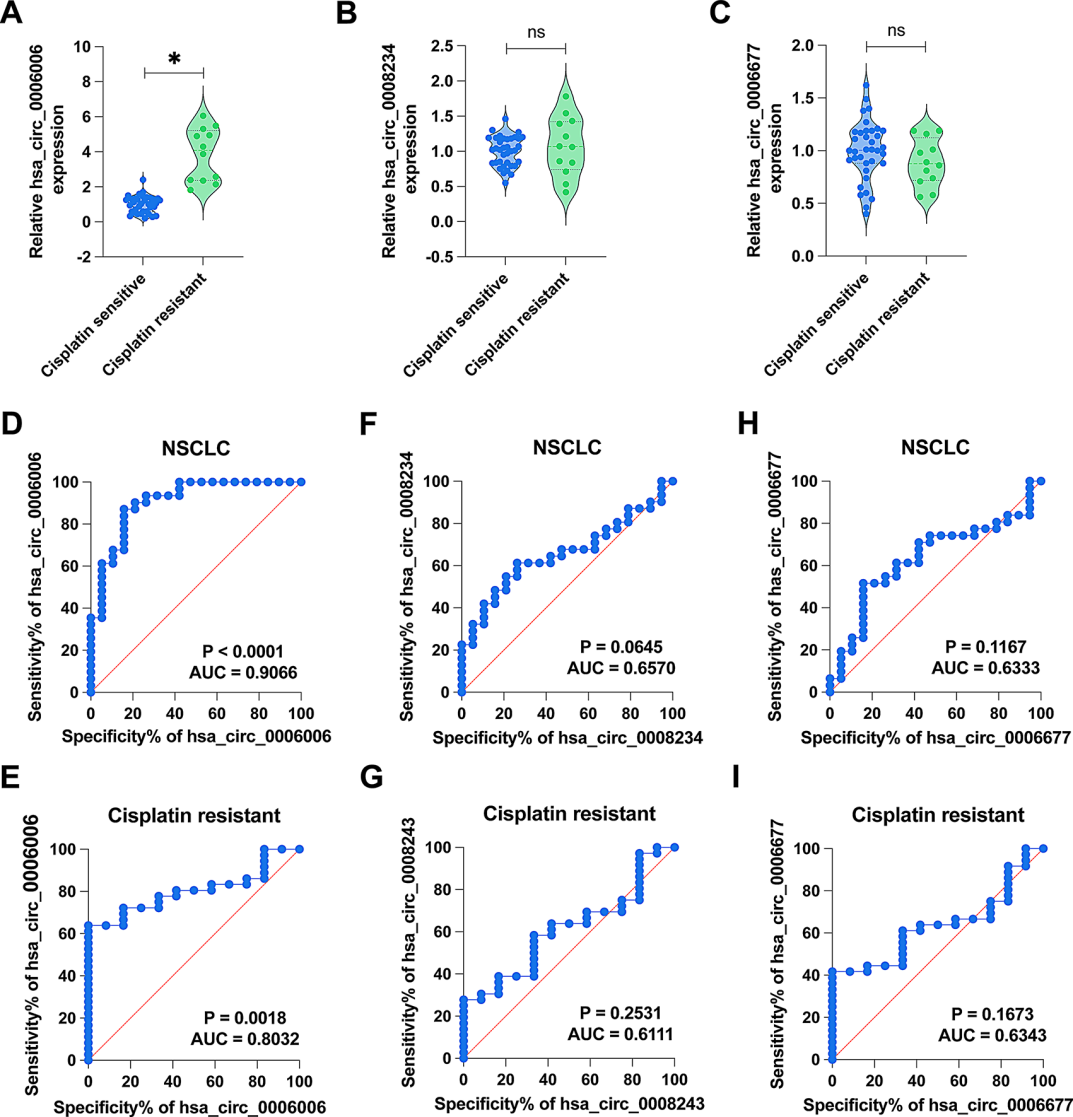
hsa_circ_0006006 and hsa_circ_0008234 can be used as clinical diagnostic markers for NSCLC. **A**–**C** RT-qPCR to detect the expression of hsa_circ_0006006, hsa_circ_0008234 and hsa_circ_0006677 in serum of DDP-sensitive and DDP-resistant patients; **D**-**E** ROC curves to analyze the potential of hsa_circ_0006006 in diagnosing NSCLC as well as DDP resistance; **F**-**G** ROC curves to analyze the potential of hsa_circ_0008234 in diagnosing NSCLC as well as DDP resistance; **H**-**I** ROC curves to analyze the potential of hsa_circ_0006677 in diagnosing NSCLC as well as DDP resistance

hsa_circ_0006006 expression was increased in the serum of DDP-resistant patients compared to DDP-sensitive patients (*P* < 0.05). However, hsa_circ_0008234 and hsa_circ_0006677 levels did not differ in the two cohorts (*P* > 0.05) (Fig. 4A-C). To further explore the diagnostic role of the three circRNAs on NSCLC and its DDP resistance, we plotted ROC curves. The results showed that hsa_circ_0006006 could effectively differentiate between healthy subjects and NSCLC patients (AUC = 0.91, *p* < 0.01) as well as DDP-resistant and DDP-sensitive patients (AUC = 0.80, *p* = 0.00) with high specificity and sensitivity (Fig. 4D-I).

### hsa_circ_0006006 is a stable circrna

**Fig. 5:**
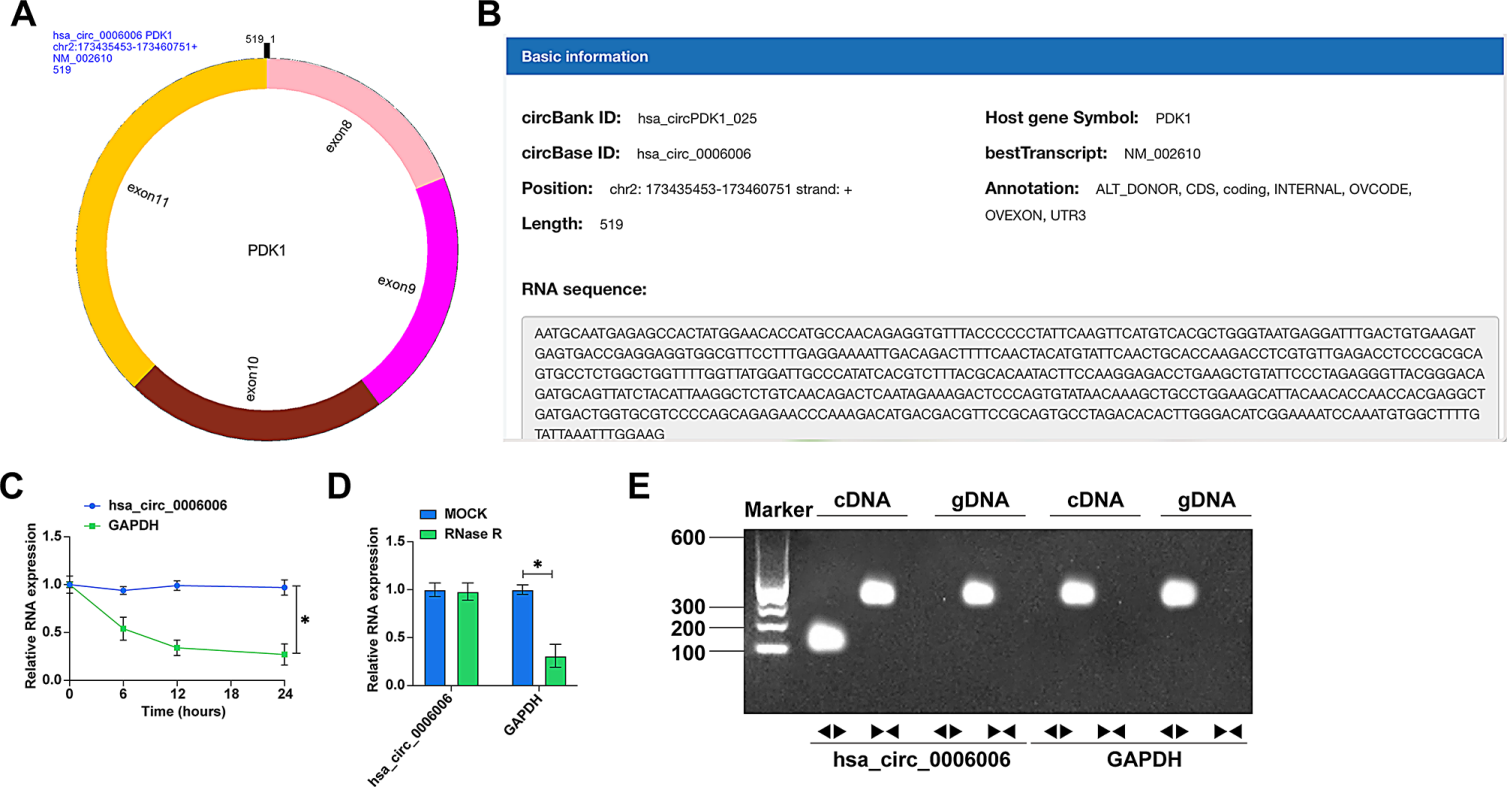
hsa_circ_0006006 is a stable circRNA. **A** CircPrism software to predict the circular structure of hsa_circ_0006006; **B** Bioinformatics website circbank to query the gene information of hsa_circ_0006006; **C** Actinomycin D experiment to detect the circular structure of hsa_circ_0006006; **D** RNase R experiment to detect the resistance of hsa_circ_0006006 resistance to RNase R digestion; **E** Gel electrophoresis to detect the ring structure of hsa_circ_0006006; data are expressed as mean ± SD (*N* = 3). * *P* < 0.05

circPrism software and bioinformatics website circbank (http://www.circbank.cn/index.html) found that hsa_circ_0006006 is composed of exons 8–11 of PDK1 located at chr2: 173,435,453–173,460,751 strand: +, with a length of 519 bp (Fig. 5A, B). Actinomycin D did not affect the stability of hsa_circ_0006006, but significantly inhibited the stability of GAPDH mRNA (Fig. 5C). RNase R could not digest hsa_circ_0006006, but could digest GAPDH mRNA (Fig. 5D). To ensure that hsa_circ_0006006 was spliced from end to end rather than trans-spliced or genomic rearrangement, divergent and convergent primers were designed. PCR results showed that hsa_circ_0006006 was only detected in cDNA, ruling out its presence in gDNA, whereas amplification of hsa_circ_0006006 and GAPDH was possible in both cDNA and gDNA using convergent primers (Fig. 5E).

### hsa_circ_0006006 enhances DDP resistance in A549 cells

**Fig. 6:**
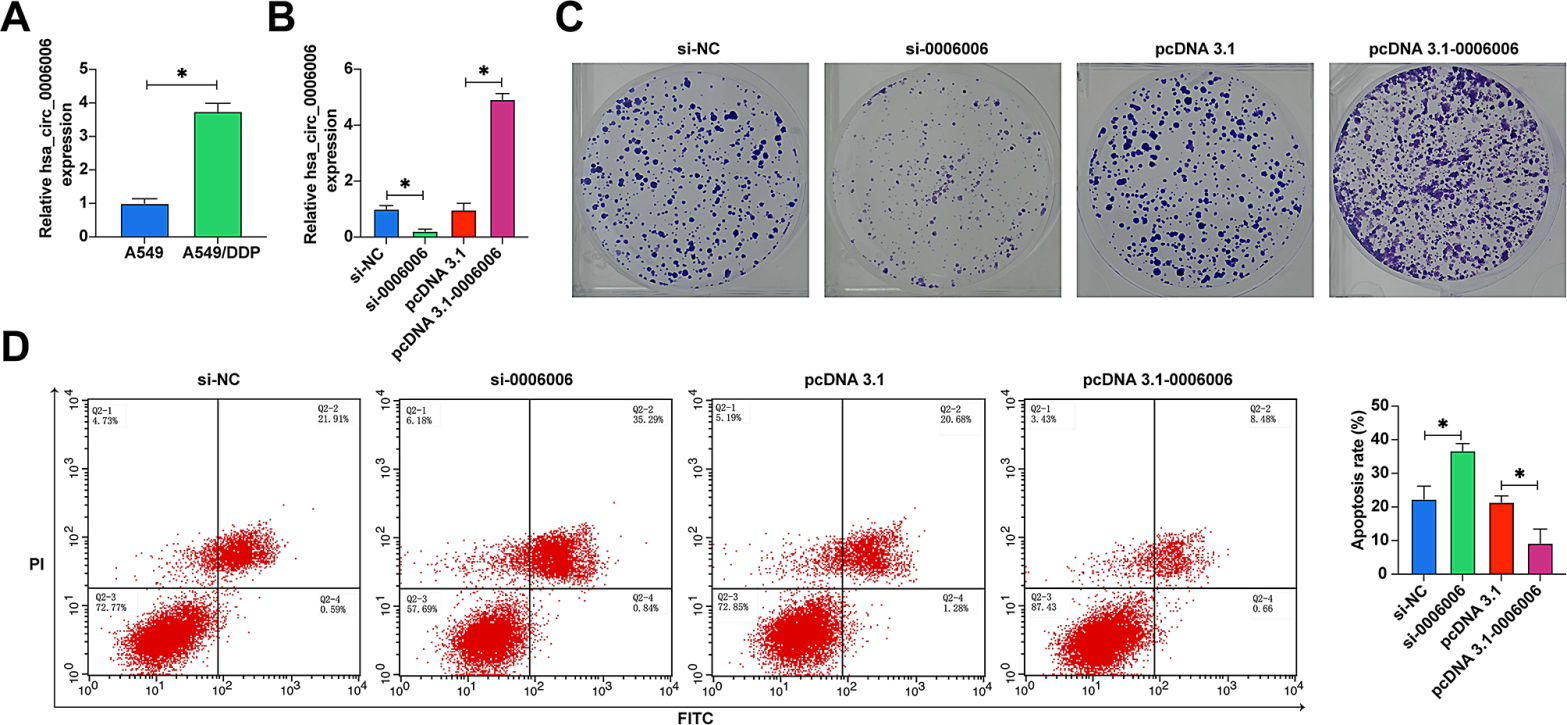
hsa_circ_0006006 enhances DDP resistance in A549 cells. **A** RT-qPCR to detect the expression of hsa_circ_0006006 in A549 and A549/DDP cells; **B** RT-qPCR to detect the transfection efficiency of siRNA and pcDNA 3.1 overexpression vectors; **C** Colony formation assay to test the proliferative ability of A549/DDP cells; **D** Flow cytometry to test the apoptosis rate of A549/DDP cells.; data are expressed as mean ± SD (*N* = 3). * *P* < 0.05

hsa_circ_0006006 was overexpressed in A549/DDP cells compared to A549 cells (Fig. 6A). hsa_circ_0006006 was knocked down and overexpressed using siRNA and pcDNA 3.1 overexpression vector, respectively (Fig. 6B). Colony formation experiments showed that knocking down hsa_circ_0006006 decreased the number of cloned cells in A549/DDP cells, but overexpressing hsa_circ_0006006 significantly enhanced cellular colony-forming ability (Fig. 6C). Flow cytometry showed that suppressing hsa_circ_0006006 increased the apoptosis rate of A549/DDP cells, but overexpressing hsa_circ_0006006 induced cellular apoptosis (Fig. 6D).

## Discussion

Research into diagnostic markers, DDP resistance markers, and survival prognostic markers encounters several obstacles and limitations in the clinical treatment of NSCLC [21]. Despite the identification of numerous molecular markers related to NSCLC, including EGFR mutations and ALK fusions, their expression is highly heterogeneous across patients and tumor subtypes, limiting their general applicability [22]. Additionally, there is a need to improve the sensitivity and specificity of existing biomarkers, especially for early NSCLC diagnosis. Despite identifying several potential mechanisms for DDP resistance, the molecular basis of this resistance is intricate and diverse [23]. In this context, the study of circRNAs offers new possibilities. Due to the tissue specificity and stability of circRNA expression in tumors, they are expected to be sensitive and specific biomarkers [10].

In this study, GO analysis of differentially expressed circRNAs revealed that they may influence tumor cell behaviors through specific biological processes and molecular functions. The results of these analyses highlight the importance of ER-PM contact sites and the regulation of protein serine/threonine kinase activities in tumor development. ER-PM contact sites serve as key hubs for intracellular signaling and are involved in the regulation of calcium signaling, lipid metabolism, and cellular stress responses [24]. The circRNAs may affect tumor cell proliferation, migration and apoptosis by regulating the expression or function of proteins associated with these contact sites. Meanwhile, the protein serine/threonine kinases play a central role in the regulation of cell cycle and proliferation [25, 26]. The inhibition of miRNAs by circRNAs through their sponging effect may indirectly enhance the function of these kinases, further affecting the growth and proliferation of tumor cells [27, 28]. circRNAs-miRNAs axis may indirectly enhance the function of these kinases and further affect the growth and proliferation of tumor cells [29]. These findings provide new insights into the molecular mechanisms of circRNAs in tumor development and may guide future therapeutic strategies against tumors.

Of the top 5 upregulated and downregulated circRNAs in the sequencing results, we found that only 3 of them had the same differential expression in the serum of NSCLC patients. This result points to a potential concordance problem between sequencing and PCR. The complexity of bioinformatics processing and sequencing data analysis may lead to discrepancies between sequencing results and RNA expression levels in biological samples. In addition, the stability and dilution of circRNAs in body fluids may have also affected the results of PCR validation. CircRNAs may be present in large amounts in tissues, but their detection in serum can be hindered by degradation or dilution. Notably, for those circRNAs (including hsa_circ_0006006, hsa_circ_0008234, and hsa_circ_0006677) that show consistency in both sequencing of cancer tissues and serum PCR assays. They may be more stable and reliable candidate biomarkers for NSCLC diagnosis, prognosis assessment or treatment response monitoring.

Previous works have demonstrated that circPDK1 is an oncogene. For example, circPDK1 competitively binds to miR-4731-5p to mediate GIGYF1 expression and increases sensitivity to paclitaxel in NSCLC [19]. Up-regulating circPDK1 promotes renal cancer cell migration and invasion [30]. Although PDK1, as a parental gene, can form multiple circRNAs, it is not evident which circRNA is functioning. In this study, hsa_circ_0006006 was highly correlated with TNM stage and lymph node metastasis in NSCLC patients. TNM stage and lymph node metastasis are important indicators of tumor prognosis [31, 32]. The association of hsa_circ_0006006 expression with these clinical parameters suggests that it can be used as a biomarker to predict the prognosis and disease progression of patients with NSCLC. hsa_circ_0008234 was only associated with TNM stage, suggesting that it may be associated with tumor size or local invasion, but not with distant metastasis. This selective association may indicate a specific role for hsa_circ_0008234 in early stages of tumors, making it a potential clinical diagnostic tool to assess local progression of tumors. Even though hsa_circ_0006677 did not demonstrate clinical significance in this study, it might still be involved in other biological processes or various tumor types.

The ROC curve analysis demonstrated that hsa_circ_0006006 could effectively distinguish between healthy individuals and NSCLC patients, as well as between DDP-resistant and DDP-sensitive individuals. The marked rise in hsa_circ_0006006 levels in DDP-resistant A549/DDP cells, along with its impact on colony formation and apoptosis rate, suggests a functional role for hsa_circ_0006006 in promoting DDP resistance. These results suggest possible targets for tailoring NSCLC patient treatments by adjusting hsa_circ_0006006 to enhance DDP response and possibly improve chemotherapy efficacy. Through the use of intervention strategies targeting hsa_circ_0006006, leading to more precise medical interventions. In addition, this study extends our understanding of the role of circRNAs in tumor chemoresistance and provides a scientific basis for the development of new therapeutic strategies.

There are also some limitations to this study. Only one NSCLC cell line was used in this study, so there are some limitations in generalizing to other NSCLC cell lines or different types of cancer cell lines. Experiments to detect hsa_circ_0006006 in other NSCLC cell lines should be added in the future to improve the credibility of the results. Second, GAPDH was used as a reference gene for gene expression data normalization in this study, but GAPDH can exhibit some variability. Although GAPDH alone was fully validated as a reference gene in our preexperiment, we will explore the options to optimize GAPDH variability in the future to provide more accurate and reliable experimental results.

In conclusion, we revealed the possible role of hsa_circ_0006006 in regulating key biological processes and signaling pathways in NSCLC. These findings provide potential new biomarkers and therapeutic targets for the diagnosis, prognostic assessment, and targeted treatment of NSCLC. However, the specific role of hsa_circ_0006006 on NSCLC and the underlying molecular mechanisms require further in-depth validation.

## Data Availability

The datasets used and/or analyzed during the present study are available from the corresponding author on reasonable request.
